# DRAI²Ned Q and A: A Comprehensive Approach to Patient Counseling

**DOI:** 10.7759/cureus.88587

**Published:** 2025-07-23

**Authors:** Gabrielle Aluisio, Alison Schultz, Tom Lindsey

**Affiliations:** 1 Medicine, Edward Via College of Osteopathic Medicine (VCOM) - Carolinas, Spartanburg, USA; 2 Simulation and Technology, Edward Via College of Osteopathic Medicine (VCOM) - Carolinas, Spartanburg, USA

**Keywords:** holistic patient care, humanism, patient-centred care, simulation center, simulation in medical education, standardized patient

## Abstract

Effective, patient-centered communication often declines over the course of medical training, particularly during standardized patient (SP) encounters. To address this gap, the DRAI^2^Ned Q&A mnemonic was created to address the critical need for patient-centered communication in medical education, particularly during SP encounters. DRAI^2^Ned Q&A systematically guides medical students through essential communication components, including diagnosis explanation, reasoning, patient knowledge assessment, shared decision-making, as well as addressing questions and creating a mutually-agreeable plan. It emphasizes empathy, patient education, and alignment of care plans, fostering collaboration and understanding. Unlike prior frameworks, it uniquely incorporates steps for clarifying misconceptions, addressing patient emotions, and gaining agreement on care plans. Although limitations like time constraints exist, DRAI^2^Ned Q&A has the potential to enhance SP performance and real-world patient satisfaction. With further validation, it can become a vital component of medical curricula, equipping future physicians with essential communication skills.

## Introduction

Incorporating humanism into medical education remains a pressing need. Studies indicate that empathy declines during medical school and residency, likely due to the high stress of mastering an abundance of medical knowledge [1,2]. One area of potential stress is standardized patient (SP) encounters. Although SP encounters are intended to evaluate communication and humanism, the medical knowledge necessary to complete them can be stressful for the learner. The general medical education curriculum may lack tools or structured instruction to guide students in effectively communicating diagnoses and care plans tailored to individual patients. This is an issue, as patient communication is a fundamental skill expected of future physicians. Although some efforts have been made to establish courses centered on humanism in medicine, additional strategies are needed to integrate these principles into the holistic training of medical students [2]. SP encounters, therefore, present as opportunities to reinforce and build humanistic approaches.

Medical education must adapt to meet contemporary healthcare demands, including frameworks that expedite recall of necessary patient-care elements. Current mnemonics taught in medical education for clinical use emphasize general care components but lack specific prompts for addressing patient-centered aspects like patient understanding and emotional responses to their diagnosis. For example, MOTHRR is a mnemonic taught in medical school to guide the plan portion of a Subjective, Objective, Assessment and Plan (SOAP) note and can be broken down into Medication, OMM, Tests to order, Humanistic/Holistic, Referral, and Return plan. Specifically, the “H” stands for the collective term “humanism,” but does not include specific prompts that medical students should ask. Thus, developing a mnemonic that integrates researched patient satisfaction methods into concise prompts will be of benefit to medical students. This will encourage medical students to recall vital patient-centered topics, such as the patient’s understanding of their diagnosis, when conducting SP encounters. To address this need, this study proposes the DRAI^2^Ned Q&A acronym, designed to guide medical students through a structured, patient-centered approach during SP encounters.

Medical frameworks have proven effective in improving interprofessional communication in healthcare. SBAR, for example, is organized into Situation, Background, Assessment, and Recommendation and emphasizes the standardization of critical information transfer during handoffs [3]. Studies have shown improved teamwork, communication, and nursing satisfaction scores utilizing SBAR among nurse practitioners and registered nurse handoffs in the emergency department [4]. Similarly, a mnemonic for structuring admission orders underscores the importance of clarity and consistency in inpatient care management. Admission orders have many facets important to acknowledge in a handoff to inpatient care, such as Admit, Diagnosis, Condition, Vitals, Activity, Nursing instructions, Diet, Allergies, Labs, IV fluids, Specialists, Medications, and Monitoring, which is appropriately summarized in the mnemonic ADC VANDALISM [5]. These tools address different aspects of healthcare communication but highlight the efficacy of structured approaches in enhancing understanding and reducing errors.

## Technical report

Methods

To design an effective mnemonic, the criteria required for Osteopathic Medical Students (OMS)-I and OMS-II at Edward Via College of Osteopathic Medicine (VCOM) during the counseling phase of SP encounters were compiled. These criteria encompassed several key elements, including pertinent findings, next treatment steps, and humanism. Pertinent findings refer to presenting symptoms, physical exam results, diagnostic outcomes, negative findings, and etiology. These are necessary for medical students to explain to their patients in an SP encounter as evidence of the patient’s diagnosis, etiology, and differentials. Next treatment steps outline physician orders such as labs, imaging, consultations, and medication adjustments. Humanism emphasizes the integration of patient-specific factors such as family dynamics or financial considerations, and that addressing patient questions ensures clarity and understanding.

A comprehensive literature review was performed to identify existing acronyms that focus on patient-centered counseling in medical education. This yielded no acronyms fitting our criteria. Additionally, the literature search was used to identify published methodologies to improve patient satisfaction and provider communication when a dismal diagnosis is given. The ABCDE acronym - which stands for Advance preparation, Build a therapeutic environment/relationship, Communicate Well, Deal with patient and family reactions, and Encourage and validate emotions- discusses the emotional aspect of delivering bad diagnoses but does not focus on the objective facts of the patient’s condition or discuss methodologies to patient satisfaction [6]. SPIKES - Setting up the interview, assessing the patient's perception, obtaining the patient's invitation, giving knowledge and information to the patient, addressing the patient's emotions with empathetic responses, and strategy and summary- is a well-established model for delivering bad news that focuses on the patient’s perception of their diagnosis. It incorporates empathy but also reminds the physician to prioritize the patient’s understanding and feelings rather than emotionally overloading the patient, but does not comprehensively cover patient-centered care components [7,8]. Similarly, SBAR - Situation, Background, Assessment, Recommendation - is a highly effective interprofessional tool but does not address direct patient interaction [3]. Additionally, “Delivering bad news to patients” isolates the physician’s discussion of a diagnosis with their patient into phases that take the patient logically through each component of their diagnosis [9]. These findings further support the need to develop a novel mnemonic tailored to SP encounters, integrating comprehensive patient-centered principles. 

The DRAI^2^Ned Q&A mnemonic was synthesized by incorporating researched and successful methods of patient satisfaction while also aligning established methodologies with VCOM’s inclusion criteria. 

Creation of DRAI^2^NED Q&A

The DRAI^2^Ned Q&A mnemonic provides a chronological, patient-centered framework for medical students during an SP encounter. The acronym is illustrated in Table 1. Each component of the acronym emphasizes essential aspects of effective communication. “Diagnosis” involves providing a clear explanation of the medical condition, while “Reasoning and risk factors” elaborates on how the diagnosis was determined and highlights relevant patient history. “Assess Knowledge” engages the patient by gauging their understanding of the diagnosis, fostering dialogue and collaboration. “Invitation” empowers patients by involving them in care decisions, reinforcing shared decision-making principles. “Information sharing” educates the patient about the disease’s etiology and dispels misconceptions. Once all the information has been shared, “Next steps” will be discussed and can be broken down into further examination and destination. “Examination” includes any further testing, appointments, consultations, etc., that will need to be placed. “Destination” is an often overlooked step in SP encounters, as it is important for medical students to inform the patient where they are going following the conclusion of the visit. Finally, “Questions” and “Agreement” ensure patient inquiries are addressed, fostering a sense of understanding and agreement of the proposed plan between the medical student and the patient.

**Table 1:** DRAI²Ned Q&A components and explanation

Components	Explanation
D	Diagnosis
R	Reasoning/risk factor: Pertinent positive and negatives from labs, patient history, physical examination, clinical presentation, etc. that confirm diagnosis
A	Assessing knowledge: What do you know about diagnosis?
I	Invitation: Would you like to discuss what caused this and your next steps?
I	Information sharing: Explain etiology of disease in a patient-centered approach that leads to current presenting symptoms. Discuss and dispel any misconceptions they have about diagnosis as discovered in Assessing Knowledge stage.
N	Next steps: Broken down into examination and destination
E	Examination: Any further labs, imaging, consultations, orders (ex: NPO) that will be ordered.
D	Destination: Where are they going? “You will be transferred to the ICU.”
Q	Questions: Do you have any questions for me?
&	And
A	Agreement: Do you agree to this plan? Is there anything I can do to assist you in this plan? “Do you have any matters at home (ex: pets) that need to be taken care of?”

Table 2 compares DRAI^2^Ned Q&A to two commonly employed methodologies in medical education for systematically delivering distressing information with empathy. SPIKES, a widely recognized acronym, is crafted for communicating distressing information, particularly for cancer patients [8,9]. Additionally, “How to Break Bad News: A Guide of Healthcare Professionals” (HTBBB) outlines five phases that physicians can follow to prepare for and deliver challenging information [6,10]. While these methodologies share a focus on the process of delivering bad news, it is important to note that they do not prescribe specific content for communicating with the patient.

**Table 2:** Components of patient communication methodologies compared to DRAI²Ned Q&A

SPIKES	How to Break Bad News: A Guide for Healthcare Professionals	DRAI^2^NED Q&A
Setting Up the Interview	Phase 1: Preparation. Establishing appropriate space, communicating time limitations, being sensitive to patient needs, being sensitive to cultural and religious values.	-
Assessing the Patient’s Perception	Phase 2: Information Acquisition. What the patient knows, how much the patient wants to know, and what the patient believes about his or her condition.	Diagnosis Reasoning Assessing Knowledge
Obtaining the Patient’s Invitation, Giving Knowledge and Information to the Patient	Phase 3: Information Sharing. Reevaluating the agenda and teaching.	Invitation Information Sharing
Addressing the Patient’s Emotions With Empathic Responses	Phase 4: Information Reception. Assessing the information reception, clarifying any miscommunication, and handling disagreements courteously.	Next Steps Further Examination Destination Questions
Strategy and Summary	Phase 5: Response. Identifying and acknowledging the patient’s response to the information and closing the interview.	Agreement

The first step in SPIKES is setting up the interview. This includes choosing the proper setting, such as a quiet conference room, demonstrating good listening skills, and focusing on the patient. The first step of HTBBB is Preparation, where patient needs are similarly established in an appropriate space. Since DRAI^2^Ned Q&A was developed for the purposes of SP encounters, the patient is assumed to be in an appropriate, safe space. Therefore, this phase in both aforementioned methodologies is not addressed in DRAI^2^Ned Q&A.

The second steps of SPIKES and HTBBB are both patient-centered and allow the patient to control the circumstances regarding their diagnosis. The “P” in SPIKES represents assessing the patient’s perception before launching into a description of the plan of care with the patient. This allows a physician to ask open-ended questions to understand how the patient perceives the medical situation. Similarly, the second phase of HTBBB is titled “information acquisition,” which details understanding what the patient knows, how much the patient wants to know, and what the patient believes about his or her condition. Although this approach is more suitable for a real-life encounter, DRAI^2^Ned Q&A is designed for a SP encounter. Therefore, in DRAI^2^Ned Q&A, the diagnosis and reasoning are included, as well as assessing the patient’s knowledge and perception of the condition. 

Step 3 and Phase 3 in SPIKES and HTBBB, respectively, have some variation in methodology. SPIKES describes Step 3 as “obtaining the patient’s invitation.” This involves the clinician waiting for the patient to explicitly express a desire for more information. Although this method is not feasible in an SP encounter, it is important to show respect for the patient's autonomy regarding their diagnosis. Phase 3 in HTBBB details information sharing regarding how much the patient desires to know. In this phase, it is important for physicians to not only divulge information, but also for the physician to listen, hear, and respond to the patient’s reactions to the information. Similarly, Step 4 of SPIKES includes giving knowledge and information to the patient. DRAI^2^Ned Q&A combines both of these methodologies into one letter. Both invitation and information sharing are equally important, as emphasized by “I^2^.” 

Step 5 and Phase 5 of SPIKES and HTBBB, respectively, involve addressing the patient’s emotions and assessing the information reception. SPIKES details addressing the patient’s emotions with empathetic responses. An empathetic response is detailed in four steps, including observing emotion in the patient, identifying the emotion, identifying the reason for the emotion, and letting the patient know you have connected the emotion with the reason for that emotion. HTBBB details the information reception phase as assessing the information reception, clarifying miscommunication, and handling disagreements courteously. DRAI^2^Ned Q&A summarizes these methodologies into Next Steps and Questions. Next Steps are further divided into further examination and destination, which gives the patient direction in their treatment plan. Additionally, Questions allow the physician to identify misconceptions and clear any miscommunications the patient may have regarding their diagnosis.

Finally, the last steps involve Strategy and Summary. SPIKES finalizes its methodology by leaving a patient with a clear plan for the future and allowing the patient time for such a discussion. Phase 5 in HTBBB involves the response of the physician, detailed by identifying and acknowledging the patient’s response to the information and closing the interview. DRAI2Ned Q&A fits summarizing and acknowledging the patient’s response in the “Question” phase discussed in the prior steps. Additionally, DRAI2Ned Q&A goes one step further by asking the patient if they agree to the plan. This incorporates alignment of patient and physician priorities, which can increase compliance.

## Discussion

DRAI^2^Ned Q&A addresses a critical gap in medical education by providing a structured framework that enhances patient-centered communication in SP encounters. Integrating components of established methodologies ensures a logical, empathetic, and collaborative approach to diagnosis delivery and care planning. The mnemonic’s patient-centered communication fosters understanding, emotional support, and engagement in care decisions while including all components necessary for a comprehensive patient treatment plan. Additionally, once priorities are aligned between a patient and their physician, treatment plan compliance may increase [11,12].

Limitations include the scope of the patient population and time constraints. Although prevalent medical methodologies were followed for delivering bad news, it is acknowledged that this approach may not be optimal for everyone and may not apply to every diagnosis. An additional limitation may be time constraints, as practicing physicians may not be able to thoroughly address each component, leading to less than optimal patient satisfaction. This could be addressed by discussing DRAI^2^Ned Q&A with practicing physicians in South Carolina in terms of practical applicability to counsel patients.

Future research will evaluate the impact of DRAI^2^Ned Q&A on SP encounter performance in the improvement of the quality of patient-centered care among OMS-I students at VCOM-Carolinas. A mixed-methods study utilizing quantitative and qualitative analysis to evaluate communication effectiveness and to assess empathy and patient satisfaction has been designed. Expanding the DRAI^2^Ned Q&A application to clinical settings and gathering feedback from practicing physicians could further refine and validate the framework as well.

## Conclusions

The development of DRAI^2^Ned Q&A fulfills an unmet need in medical education by offering a structured, patient-centered mnemonic tailored to SP encounters. By synthesizing principles from existing frameworks and incorporating patient-specific prompts, it equips medical students with essential communication skills. With further validation, the framework has the potential to enhance patient satisfaction and care quality, supporting its integration into medical school curricula. DRAI^2^Ned Q&A may improve provider-patient interactions by providing medical students early on with a patient-centered, yet comprehensive, framework as they counsel SPs. It is an ideal candidate to be implemented into the medical curriculum to prepare students for future patient interactions as practicing physicians.
